# Predicting treatment outcome using kinome activity profiling in HER2+ breast cancer biopsies

**DOI:** 10.1016/j.isci.2024.109858

**Published:** 2024-04-30

**Authors:** Donna O. Debets, Erik L. de Graaf, Marte C. Liefaard, Gabe S. Sonke, Esther H. Lips, Anna Ressa, Maarten Altelaar

**Affiliations:** 1 Biomolecular Mass Spectrometry and Proteomics, Bijvoet Center for Biomolecular Research and Utrecht Institute for Pharmaceutical Sciences, University of Utrecht, Utrecht, 3584 CH Utrecht, the Netherlands; 2 Pepscope B.V, Nieuwe Kanaal 7, 6709 PA Wageningen, the Netherlands; 3 Department of Molecular Pathology, The Netherlands Cancer Institute, Amsterdam, the Netherlands; 4 Department of Medical Oncology, The Netherlands Cancer Institute, Amsterdam, the Netherlands; 5 Department of Medical Oncology, University of Amsterdam, Amsterdam, the Netherlands

**Keywords:** Oncology, molecular biology, cancer, proteomics

## Abstract

In this study, we measured the kinase activity profiles of 32 pre-treatment tumor biopsies of HER2-positive breast cancer patients. The aim of this study was to assess the prognostic potential of kinase activity levels, to identify potential mechanisms of resistance and to predict treatment success of HER2-targeted therapy combined with chemotherapy. Indeed, our system-wide kinase activity analysis allowed us to link kinase activity to treatment response. Overall, high kinase activity in the HER2-pathway was associated with good treatment outcome. We found eleven kinases differentially regulated between treatment outcome groups, including well-known players in therapy resistance, such as p38a, ERK, and FAK, and an unreported one, namely MARK1. Lastly, we defined an optimal signature of four kinases in a multiple logistic regression diagnostic test for prediction of treatment outcome (AUC = 0.926). This kinase signature showed high sensitivity and specificity, indicating its potential as predictive biomarker for treatment success of HER2-targeted therapy.

## Highlights

• Measured kinase activity in 32 pre-treatment HER2-positive BC patient tumor biopsies
• The acquired activity profiles connect kinase activity to treatment response
• High kinase activity in the HER2-pathway is associated with good treatment outcome
• Kinases signature shows potential as predictive biomarker for HER2-targeted therapy

## Graphical abstract

**Figure:**
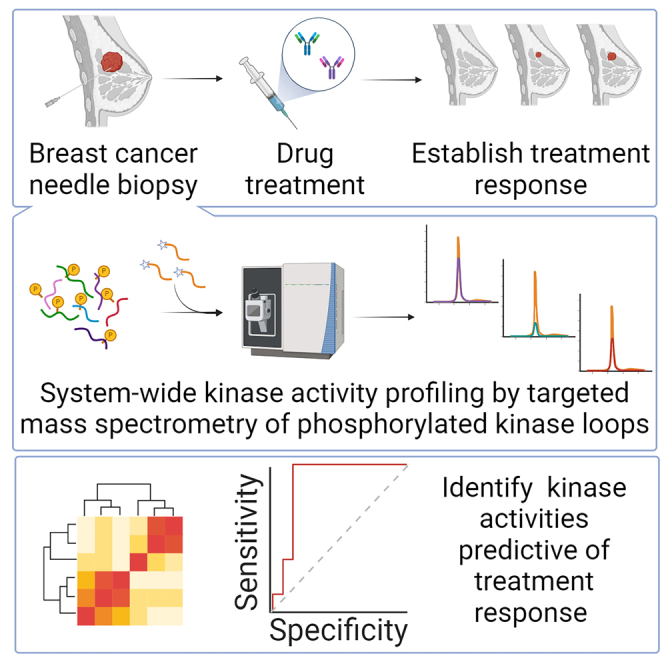

## Introduction

Invasive breast cancer (IBC) is a highly heterogeneous disease, which is classified in subtypes with distinct molecular signatures.^1^ The disease course, survival rate and treatment strategy is highly dependent on the subtype. Around 15% of all breast cancer cases overexpress the human epidermal growth factor receptor 2 (ERBB2 or HER2) and is therefore referred to as HER2+ (HER2 positive) breast cancer. This subtype is more aggressive and typically has a poor treatment outcome.^2^^,^^3^^,^^4^ The development of antibodies blocking the HER2 receptor (Trastuzumab, TTZ, and Pertuzumab, PTZ) have improved the clinical outcome of HER2+ patients profoundly.^4^^,^^5^^,^^6^^,^^7^ Consequently, these drugs have found their way to standard of care.^8^^,^^9^

Treatment resistance against HER2-inhibition (both primary and acquired) is observed frequently.^10^ The development of treatment resistance has been extensively studied in the last decade resulting in the discovery of a multitude of different resistance mechanisms.^10^^,^^11^ Despite these discoveries, predictive biomarkers for treatment success are still absent, hampering effective clinical decision-making.

Many of the proposed resistance mechanisms evolve around rewiring of cellular signaling pathways. This can be either due to activation of alternative survival routes bypassing the HER2-pathway or the re-activation of HER2 and/or downstream signaling nodes via compensatory, redundant or mutated signaling molecules. The myriad of potential escape mechanisms demonstrates the need for patient-specific treatment strategies and hence appropriate patient stratification. However, the lack of effective diagnostic tools to pinpoint the rewiring mechanisms in a case-by-case fashion in a clinically relevant setting hampers biomarker identification and development of precision medicine.

Several strategies exist to measure rewiring of cellular signaling pathways; however, none of them provide all the relevant information. Since cellular signaling is heavily dependent on rapid and reversible protein phosphorylation,^12^^,^^13^ gene-, transcript-, and protein-based analysis are insufficient. These techniques provide insights into pathway alterations, yet fail to pinpoint pathway activation. Phosphoprotein analysis, such as phosphoprotein-specific western blots and discovery phosphoproteomics, provide a more detailed view on which kinase substrates are activated within a pathway. Antibody-based techniques are generally very sensitive, yet their use is restricted by the limited availability of phosphosite-specific antibodies. Furthermore, due to its poor multiplexing capabilities antibody-based studies are generally focused on a few phosphosites, providing limited pathway coverage and only allow for hypothesis-driven research. Phosphoproteomics-based techniques provide a more system-wide view, covering multiple signaling pathways in a single analysis, making it ideal to monitor signal transduction rewiring and to find tumor resistance mechanisms. However, determination of the exact kinase responsible for pathway rewiring is hampered by information-bias of kinase-substrate pairs and kinase motifs.

To overcome these limitations, an alternative type of phosphoproteomics technology was developed previously, which allows for the direct measurement of kinase and pathway activity in a high-throughput and precise manner, covering a large set of kinases (currently, one-third of the total kinome).^14^ This technology, QuantaKinome, is based on measurement of the phosphorylation of kinase activation loops (T-loops), which in the majority of cases is a direct proxy for kinase activity.^15^ The precise quantification of such a large panel of kinase activities provides a comprehensive understanding of pathway activation and cellular rewiring in a simple, fast, and precise way. Furthermore, since this approach does not rely on prior knowledge on substrate phosphorylation as surrogate for kinase activity, it allows for the discovery of the role of “dark” or understudied kinases. Lastly, the unbiased system-wide approach grants the possibility of hypothesis-generating discovery studies.

In the current study, we applied this kinase activation loop assay to 32 treatment-naive HER2+ breast cancer biopsies to identify kinases and pathways linked to treatment success and to improve patient stratification. We found that increased HER2-pathway signaling was associated with treatment success. Furthermore, we identified 11 kinase activation states that were differentially regulated between treatment outcome groups. Lastly, we defined a panel of 4 kinases that were predictive of treatment outcome; the high specificity and sensitivity of this panel illustrates the potential of kinase activity as a predictive biomarker for treatment success of HER2-targeted therapy.

## Results

To identify kinase activities predictive of treatment success, we performed Quantakinome analysis on 32 treatment naive breast cancer biopsies. These clinical samples originated from the TRAIN-2 randomized phase 3 clinical trial^16^^,^^17^ in which treatment-naive patients with stage II–III HER2+ breast cancer tumors were enrolled. All patients received therapy consisting of dual HER2 blockade (TTZand PTZ) in combination with varying types of chemotherapy (with or without anthracyclines). The clinical outcome was defined by a pathologist as pathological complete response (pCR, no tumor cells left), near pathological complete response (npCR; <10% of cells contained invasive tumor cells) and no pathological complete response (No pCR; >10% tumor cells remaining). Histological image representation of the three treatment outcome groups is shown in Figure S1. Patients classified as No pCR are referred to as treatment resistant throughout this study. Prior to drug treatment, a 14-G core needle biopsy was taken for local histology-based assessment and the remainder was stored for later analysis. In the current study, we received 32 of these pre-treatment tumor biopsy samples for QuantaKinome analysis. In total, 17 patients were classified as pCR, 6 as npCR, and 9 as No pCR. A complete list of patient biopsy details is provided in Table S1.

**Figure 1:**
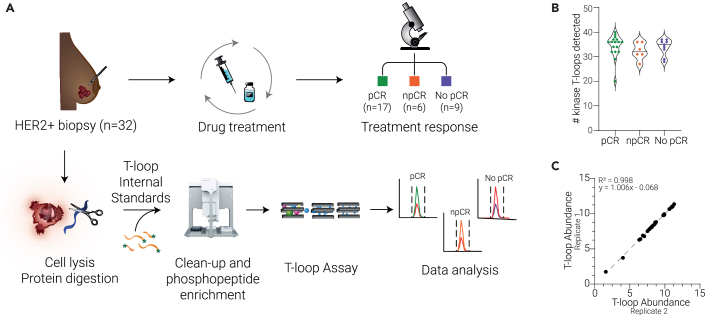
Study overview (A) Experimental workflow. (B) Number of kinase T-loops quantified per patient biopsy in each treatment outcome group. (C) T-loop abundance reproducibility of two technical workflow replicates that were created by splitting a single patient sample in two.

The tumor biopsies were lysed, and the proteins were extracted and digested before being desalted on an automated platform and spiked with an internal reference stable-isotope labeled standard for each phosphorylated kinase T-loop. Subsequently, phosphorylated peptides were enriched by automated phosphopeptide enrichment. Thereafter, the signal of 311 T-loop peptides was measured using a single targeted mass spectrometry assay on a triple quadrupole mass spectrometer. Finally, endogenous kinase T-loop signals in each sample were normalized using their respective internal standard, to achieve a precise quantification of kinase activation. The complete workflow is depicted in Figure 1A.

In total, we successfully detected 307 kinase T-loop reference standards of which 56 were endogenously quantified. Five of the endogenously detected T-loop phosphopeptides contained a methionine oxidation and 4 were doubly phosphorylated, yielding a total of 51 unique kinase activation states, which were mapped to 61 endogenous kinases (Figure S2A). The run-to-run quantitative reproducibility was high (Figure S2B). The number of quantified kinase T-loops per patient ranged from 20 to 40 and was comparable between the three treatment outcome groups (Figure 1B).

One patient biopsy sample contained enough material to perform the entire workflow in duplicate on the exact same sample allowing us to explore the reproducibility of the workflow. As shown in Figure 1C, the correlation of T-loop abundances measured in two technical workflow replicates originating from a single patient sample is very strong (R = 0.998). The high correlation coefficient together with a slope of one and an offset near zero indicates a high reproducibility of the method in clinical samples.

A high dynamic range in the detection of kinases was found; the most abundant kinase T-loop (GSK3A [Y279]) was more than 4,400-fold higher abundant compared to the lowest detected kinase T-loop (NEK6 [S206]). Figure S2C illustrates that all kinase families show a wide dynamic range, and that the top five most abundant kinase T-loops are from the CMGC-family. Moreover, some kinases within the CMGC-family show high correlation, indicating that these kinases could be co-regulated. However, as shown in Figure S2D, overall, the kinase activity does not seem to be regulated family-wide, but rather on the individual kinase level. Typically, T-loop phosphorylation sites within the same kinase (such as CDK7 and MARK1) have a high correlation.

The biological relevance of the detected kinases is evident from their high mutation frequency in breast cancer, which is similar to other well-known non-kinase breast cancer markers such as BRCA2 and ESR1 (Figure S2E). Furthermore, Figure S2F illustrates that the quantified kinase T-loops in this study consisted of both extensively studied kinases (such as ERK1/2, p38A, and JNK1/2/3) and under-studied kinases.

### Low kinase activity in HER2-pathway is associated with treatment resistance

**Figure 2:**
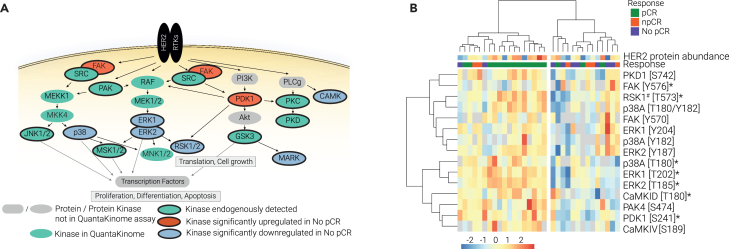
Low kinase activity levels within HER2 pathway are associated with treatment resistance (A) Graphical representation of HER2-pathway. Kinases quantified in this study are highlighted. (B) Unsupervised clustering of HER2-pathway kinase activities (Euclidean distance, Z-scored data). HER2 protein expression levels are displayed above the heatmap. ∗Significantly regulated kinase; #kinase T-loop peptide was shared with at least one paralog.

Since the targeted therapy used in this study is directed against HER2, we first explored the activity of kinases (by T-loop phosphorylation) relevant in this pathway. Firstly, the HER2-pathway was extensively covered in our dataset, including crucial kinases such as ERK, PDK1, and RSK1 (Figure 2A). Importantly, we found many kinases in this pathway differentially regulated between the tumors that were treatment resistant (no pCR) compared to the treatment responsive tumors (pCR and npCR). Most differentially regulated kinases showed a lower kinase activity in treatment resistant tumors (such as ERK1, ERK2, RSK1, CaMKID, and p38). However, one kinase was found upregulated among the resistant tumors: focal adhesion kinase (FAK).

Unsupervised clustering of key kinases within the HER2-pathway grouped patients into two main clusters. One cluster contained the majority of the treatment resistant tumors and displayed low kinase activity levels (Figure 2B). HER2 protein expression levels (displayed above the heatmap in Figure 2B) did not account for the reduced downstream signaling, highlighting the importance of the broad assessment of kinase activities. We observed a kinase activity heterogeneity within patient groups, indicating that individual patients can have different nodes activated within the HER2-pathway, leading to a similar outcome. Overall, there is consensus on the pathway level: reduced kinase activity within the HER2-pathway was associated with treatment resistance. This could indicate that these tumors were less HER2-driven and hence less susceptible to HER2-inhibition.

### Differentially regulated kinase activities are predictive of treatment outcome

**Figure 3:**
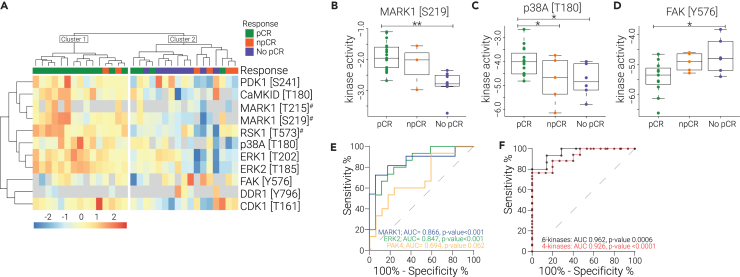
Kinase activities (by T-loop phosphorylation) differentiate treatment outcome groups (A) Heatmap of unsupervised clustering of all significantly regulated kinase T-loops (ANOVA *p* value <0.05) (Euclidean distance, data were Z-scored). # kinase T-loop was shared with at least one close paralog. (B–D) Boxplots of regulated kinase T-loops. ∗ *p* value <0.05; ∗∗ *p* value <0.001. E) Receiver operator characteristics (ROC) curve analysis to predict treatment outcome based on kinase T-loop abundance for single kinases. (F) ROC curve to predict treatment outcome based on a panel of kinases. Six-kinase panel consisted of kinase T-loops detected in at least 80% of the patients, with a minimum of 1.5-fold difference and max *p* value of 0.05. Four-kinase panel consisted of kinase T-loops detected in all patients with *p* value <0.05.

The primary goal of this study was to identify kinases that predict therapy response in HER2+ breast cancer treated with a combination of HER2-blockade and chemotherapy. An ANOVA-test revealed eleven kinases that were differentially activated between the pCR, npCR, and No pCR groups. Unsupervised hierarchical clustering of these regulated kinases revealed two clusters of patients (Figure 3A). Cluster 1 consisted of mainly pCR tumors, which displayed a high relative kinase activity. Cluster 2 contained npCR and all No pCR tumors, characterized by generally lower kinase activity levels. As discussed previously, many of these kinases were involved in the HER2-pathway.

MARK1 (microtubule affinity-regulated kinase, also known as Par-1), RSK1, CDK1, and CaMKID were significantly downregulated among treatment resistant tumors and showed intermediate levels for the npCR tumors (Figures 3A, 3B, and S3A). P38A, ERK1, and ERK2 contrarily were downregulated in both the npCR and No pCR tumors compared to the pCR tumors (Figures 3C and S3A). This suggests that p38A, ERK1, and ERK2 could be important in treatment sensitivity yet are not sufficient for a full treatment response, whereas MARK1, RSK1, CDK1, and CaMKID are stronger determinants for a complete treatment response. Apart from CDK1 and MARK1, all of these kinases were involved in the HER2-pathway highlighting that reduced HER2-pathway kinase activity was linked to treatment resistance.

Interestingly, FAK showed an opposite trend, displaying a higher activity in the No pCR tumors (Figure 3D). FAK integrates signaling events from two different types of receptors; it is a key downstream kinase of both growth factor and integrin receptors. Hence, increased FAK activity suggests that these alternative signaling routes might be important in treatment resistant tumors and that tumors could escape HER2-inhibition via this alternative signaling node.

To assess whether kinase activity levels could be used to predict treatment outcome, a receiver-operating characteristic (ROC) curve analysis was performed using both single kinase activity levels and a panel of kinases. For clinically relevant predictions two patient groups were formed; pCR patients were considered “responders”, whereas No and npCR patients were designated “non-responders”. The kinase activity differences between these two groups and corresponding ROC characteristics are tabulated in Table 1.

**Table 1:** Kinase activities regulated in clinical groups and used for ROC analysis

Kinase Activity	Log2 FC Responders vs. Non-Responders	T-test *p* value	No. Patients Detected	AUC	ROC *p* value
ERK2 (T185)	1.10	<0.001	32 (100%)	0.847	0.0008
MARK1 (S219)^a^	0.75	0.001	26 (81.3%)	0.866	0.0013
RSK1 (T573)^a^	0.89	0.003	32 (100%)	0.792	0.0049
CaMK1D (T180)	1.15	0.007	28 (87.5%)	0.797	0.0081
ERK1 (T202)	0.72	0.008	32 (100%)	0.741	0.0202
FAK (Y576)	−0.64	0.009	22 (68.8%)	0.818	0.0115
p38A (T180)	0.78	0.010	26 (81.3%)	0.764	0.0240
MARK1 (T214)^a^	1.10	0.013	13 (40.6%)	0.762	0.1161
CaMKIV (S189)^a^	0.42	0.026	24 (75%)	0.748	0.0397
p38A (T180 + Y182)	0.54	0.031	29 (90.6%)	0.733	0.0325
PAK4 (S474)	0.57	0.046	32 (100%)	0.694	0.0616
DDR1 (Y796)	−2.22	0.049	7 (21.9%)	1.000	0.0339

a phosphorylated T-loop shared with protein paralogs.

The overall performance of our diagnostic markers to predict treatment outcome was assessed using the area under the curve (AUC) of the ROC. The AUC for the individual kinase activity measurements ranged from 0.866 to 0.694 (Table 1; Figure 3E). The two most significantly regulated kinase activities (ERK2 T185 and MARK1 S219) showed good predictive potential with an AUC ≥0.85.

Previous research has shown that tumors display considerable heterogeneity between patients; tumors utilize different signaling routes to drive tumor growth or treatment evasion.^18^^,^^19^ Indeed, we also observed varying kinase activity levels between and within the patient groups in this study (Figures 2A and 3A). Therefore, to account for this patient heterogeneity in a diagnostic test, we explored the use of a panel of kinase activities to better predict treatment response. The first kinase panel consisted of 6 kinase activities (RSK1 T573, CaMK1D T180, p38A T180, ERK1 T202, ERK2 T185, and MARK1 S219) from the HER2-signalling pathway that were selected based on their *p* value (<0.05), fold change (>1.5-fold), and detectability (detected in at least 80% of patients). For the second panel, kinase activation states were only considered if they were significantly regulated and detected in all patients. This resulted in a set of 4 kinases (RSK1 T573, ERK1 T202, ERK T185, and PAK4 S474). A multiple logistic regression of both kinase signatures resulted in higher AUC values (0.962 and 0.926, respectively) compared to the best single kinase predictors (Figure 3F). In summary, the 4-kinase panel showed a strong basis for a prognostic test that performed better than the best single kinase T-loop (MARK1 S219) with an added benefit of 100% detectability compared to 81.3%.

## Discussion

The significance of phosphorylation dynamics and kinase activity in health and disease is indisputable. In this study, we applied a method previously developed by Schmidlin et al.^14^ for the direct measurement of kinase activity (as measured by kinase T-loop phosphorylation) in 32 breast cancer biopsies. Results of this technology demonstrated a high reproducibility and sampling depth from low sample quantities. We quantified 56 kinase activation states, largely covering the HER2-pathway. Classical approaches use substrate phosphorylation as proxy for kinase activation; however, determination of the exact activated kinase is hindered by information-bias and overlapping substrate specificity. Our kinase T-loop assay contrarily, directly infers kinase activity from T-loop phosphorylation.

The primary goal of this study was to explore the potential of kinase activation states in predicting therapy response in HER2+ breast cancer patients. 11 kinase activation states were significantly regulated between the different response groups. Patients that responded well to therapy showed a generally higher kinase activation profile, especially in the HER2-pathway. Poor responders displayed reduced kinase activities within the HER2-pathway, indicating a lower HER2-dependency among these tumors and hence poor response to HER2-inhibition. This is in line with previous research showing that decreased HER2-expression levels were linked to unfavourable treatment outcomes.^20^^,^^21^ Importantly, we observed heterogeneity on the individual kinase level within the HER2 pathway, highlighting the importance of a broad coverage of signaling nodes to catch tumor heterogeneity for mapping pathway activation.

The prognostic value of kinase activation levels was demonstrated by ROC analysis. Several kinase activation states were indeed predictive for treatment success of HER2-targeted therapy. Furthermore, the prognostic power improved when a panel of kinase activities was used; the benefit of this panel compared to single kinase activities likely results from the heterogeneity in activating signaling nodes between patients. Hence, combining the activation states of multiple kinases within a single diagnostic test is crucial. To determine whether this panel can be used as a real actionable biomarker a larger patient cohort is needed.

In this study, we found significant downregulation of p38A (T180) among the treatment resistant tumors. Previous research has revealed the highly complex and context-specific function of p38 in drug resistance in cancer. In leukemia, upregulation of p38 is linked to drug resistance against genotoxic chemotherapy,^22^ whereas the opposite holds true for targeted therapy; increased p38 phosphorylation is then linked to increased drug sensitivity.^23^^,^^24^ Furthermore, resistance against EGFR inhibitors in lung cancer can be overcome by dual inhibition of MEK and PI3K via activation of p38 signaling.^25^ The antibody-based techniques used as readout for p38-activation could partially explain the different roles attributed to this kinase, since distinct biological functions have been ascribed to different phosphorylation states of p38.^26^ Due to the close proximity of these phosphorylation sites in the kinase T-loop, these are difficult to distinguish using antibodies. In our study, we quantified multiple T-loop activation states of p38A, of which only p38A (T180) was found significantly changing (Figure S3B). This suggests diverse functions of p38A activation states and highlights the potential of our method to distinguish between these kinase states.

Among the treatment resistant tumors, we found a significant upregulation of FAK activity at FAK Y576. A growing body of evidence suggests that FAK may play an important role in cancer biology and therapy resistance and a variety of FAK-inhibitors is currently in development.^27^^,^^28^ FAK has been found over-expressed and/or hyper-phosphorylated in many cancers, including breast cancer.^29^^,^^30^ This over-expression or over-activation is associated with increased cell motility, survival, proliferation, and poor clinical outcome.^31^^,^^32^^,^^33^^,^^34^ Furthermore, FAK has been linked to TTZ resistance via compensatory cross-talk^30^ and inhibition of FAK has been shown to help to overcome this resistance.^35^ Our data agrees with these findings and suggests that over-activation of FAK might indeed play a role in reduced drug sensitivity. The increased FAK activity could provide alternative signaling nodes aiding in the tumor’s escape of HER2-inhibition.

In addition to the differential regulation of these well-described kinases, we also identified a significant downregulation of the relatively unexplored kinase MARK1 among treatment resistant tumors. This serine-threonine kinase is often found amplified in breast cancer (Figure S2E) and plays a role in cell motility and regulation of energy metabolism. Recently, MARK1 has been discovered as the direct target of microRNA in both cervical and colorectal cancer where it was linked to proliferation and cell migration.^36^^,^^37^ Furthermore, MARK1 is a substrate of LKB1, a well-known tumor suppressor gene linked to metastatic outgrowth of cancer cells.^38^^,^^39^^,^^40^ In this study, we link increased MARK1 activation to better treatment response and show that the activation status of this kinase is predictive for drug resistance in this cohort of patients. Since computational tools used in shotgun phosphoproteomics exploit substrate phosphorylation as surrogate for kinase activity, it is inherently biased toward well-studied kinases. Hence, a kinase such as MARK1 (for which the downstream targets are largely unknown) will remain hidden in these conventional methods. The direct measurement of kinase activity used in this study does not suffer from this information-bias and is therefore able to identify this kinase as a potential player in drug resistance.

Overall, our study shows the large potential of this technology in the prediction of treatment response *in vivo*.

### Limitations of the study

We emphasize that the suitability of the kinase T-loop assay as a diagnostic tool and its prognostic value in breast cancer treatment requires further validation in larger clinical cohorts, especially seeing the small sample size of this study. In addition, because all patients within the cohort received both chemotherapy and targeted therapy, we cannot delineate which treatment regimen correlates with our observed kinase activity profiles. We acknowledge that these results should be interpreted cautiously and require further study before they can be considered clinically actionable.

## STAR★Methods

### Key resources table

**Table:** 

REAGENT or RESOURCE	SOURCE	IDENTIFIER
**Biological samples**		

Human IBC tumor samples	TRAIN2 clinical trial	https://clinicaltrials.gov/ct2/show/NCT01996267

**Chemicals, peptides and recombinant proteins**		

Sodium deoxycholate (SDC)	Sigma Aldrich	MFCD00064139
Tris(2- carboxyethyl) phosphinehydrochloride (TCEP)	Sigma Aldrich	MFCD00145469
Chloroacetamide (CAA)	Sigma Aldrich	MFCD0008027
TRIS	Sigma Aldrich	
PhosSTOP	Merck	4906837001
cOmplete, Mini, EDTA-free Protease Inhibitor Cocktail	Merck	11836170001
Trypsin	Thermo Scientific	Prod# 90057S
LysC	Wako	125–05061
SpikeMix Kinase Activation Loops (Human) - heavy	JPT Germany	SPT-KAL-POOL-L-100p.m.
iRT Kit	Biognosys	Ki-3002-1

**Critical commercial assays**		

AssayMap Cartridge Rack, Fe(III)-NTA 5 mL	Agilent Technologies	Cat#G5496-60085
AssayMAP Cartridge Rack C18 5ul	Agilent Technologies	Cat# 5190-6532
Bradford Protein Assay	Bio-Rad	5000006
PepMap RSLC C18 2um, 100A 75x25	Thermo Scientific	ES802

**Deposited data**		

Skyline documents	PanoramaWeb	PXD035186
Raw files	PanoramaWeb	PXD035186

**Software and algorithms**		

Skyline	Skyline	https://skyline.ms/project/home/software/Skyline/
GraphPad Prism 9.3.0	Graphpad Software Inc	https://www.graphpad.com/scientific-software/prism/
R v4.3.3	https://www.r-project.org/	https://www.r-project.org/
RStudio (v2022.02.1 461)	RStudio, PBC	https://www.rstudio.com/

### Resource availability

#### Lead contact

Further information and requests for resources and reagents should be directed to and will be fulfilled by the lead contact, Maarten Altelaar (m.altelaar@uu.nl).

#### Materials availability

The study did not generate new unique reagents.

#### Data and code availability

• Raw data and processed data were uploaded to Panorama and are available via the ProteomeXchange ID: PXD035186.
• The mass spectrometry data used in this study has been deposited to Panorama.
• This study did not generate original code.
• Any additional information required to reanalyses the data reported in this paper is available form the lead contact upon request.

### Experimental model and study participant details

Patient biopsies were obtained from patients enrolled in the TRAIN-2 study.^41^ All included patients provided written informed consent. The medical ethics committee of the Netherlands Cancer Institute approved the study protocol and all amendments thereof.

Sex of study participants was female and age ≥18 years.

### Method details

#### Biopsy preparation

Patient biopsies were obtained from patients enrolled in the TRAIN-2 study.^41^ The patients had a median age of 47 years (range 29–73 years). The study was approved by the ethical committee and informed consent was obtained. Patients overexpressed HER2 and received neoadjuvant therapy consisting of Trastuzumab and Pertuzumab supplemented with either 5-fluorouracil, epirubicin, cyclophosphamide or a combination of paclitaxel and carboplatin. Prior to start of the treatment a 14G needle biopsy was taken of approximately 30 μm and flash frozen in liquid nitrogen. Part of this biopsy was used to perform hematoxylin and eosin (HE) staining to determine tumor cell content, while 1/3 of the biopsy was snap frozen and kept at −80°C for QuantaKinome analysis. Only samples with a minimum tumor percentage of 60% were used in this study. After nine cycles of drug treatment, the response was determined at surgery.

#### Kinase activity analysis using QuantaKinome

The QuantaKinome platform was applied to measure T-loop phosphopeptides by using a targeted LC-MS approach which is based on the method described in Schmidlin et al. Briefly, frozen biopsy were lysed in SDC lysis buffer, proteins were extracted and 200 μg of protein was digested with LysC for 2 h (1:50 enzyme to protein ratio) and trypsin overnight (1:50 enzyme to protein ratio) (method is described in more detail previously by Schmidlin et al.^14^). Peptides were desalted and phosphorylated peptides were enriched using an automated platform (Agilent Bravo) as described previously (Post et al.). Samples were dried and stored at −80°C until LC-MS analysis, performed on a TSQ Altis (Thermo Scientific) coupled to an Ultimate 3000 ((Thermo Scientific) equipped with a ES802 analytical LC column. Next, half of the processed samples were measured in randomized order using the targeted LC-MS assay. Samples were dissolved in 2% FA and loaded on an ES802 column on an Ultimate 3000 RSLC nano LC coupled to a TSQ Altis (Thermo Scientific).

### Quantification and statistical analysis

#### Preprocessing of datasets

Raw files were uploaded into Skyline^42^ and peak boundaries were manually checked and adjusted if needed. Subsequently, an in-house build R-script was used to filter-out endogenous peptides with interference. First, all transitions with a signal-to-noise level below three times the background were removed. Subsequently, for each peptide the relative contribution of each transition to the total peptide signal was calculated and light transitions with a difference of more than 20% compared to the heavy standard relative contribution were removed. Next, peptides with less than two transitions were removed. Lastly, peptides that were detected in less than five files were removed. Remaining peptides were visually inspected in Skyline. After peptide identification, the transitions suitable for quantification were chosen to reliably quantify the peptides over all samples. For each peptide, only transitions that showed a consistent light/heavy ratio in all samples were used. Furthermore, quantification was based on at least two transitions. The same transitions were used to quantify across all files. Lastly, a weighted and internal standard corrected T-loop abundance was created by dividing the sum of the light transitions by the sum of the heavy transitions following a log2 transformation. The T-loop abundance is also referred to as kinase activity in this manuscript.

Some peptides were detected in oxidised and non-oxidised form. Oxidised T-loop phosphopeptides were detected less frequently and generally showed a 10-fold lower signal compared to their non-oxidized counterpart (Figure S4). The correlation between the two oxidation forms was high in all samples, indicating that the oxidation rate was similar between all samples. Therefore, when both oxidation states were detected, only the non-oxidized form was used to calculate the kinase T-loop abundance level.

#### Statistics

To compare three means, a one-way ANOVA test was used (using the aov-function, followed by TukeyHSD-function in Rstudio). A *p*-value below 0.05 was regarded as statistically significant. Correlation analysis was performed by Pearson correlation using cor-function in RStudio.

Unsupervised clustering and visualization were performed in R using the pheatmap package. The data were z-scored (scaled by row) prior to clustering. Euclidean distance was used for both row and column clustering. ROC analysis and multiple logistic regression was performed in Graphpad Prism 9.

## Supplemental information

Document S1. Figures S1‒S4 and Table S1
