# Supplementary material for: Predicting treatment outcome using kinome activity profiling in HER2+ breast cancer biopsies
Source: iScience. 2024 Apr 30;27(6):109858. doi: 10.1016/j.isci.2024.109858 (PMC11112361; doi:10.1016/j.isci.2024.109858)

iScience, Volume *27*

Supplemental information

Predicting treatment outcome using kinome activity profiling in HER2+ breast cancer biopsies

Donna O. Debets, Erik L. de Graaf, Marte C. Liefaard, Gabe S. Sonke, Esther H. Lips, Anna Ressa, and Maarten Altelaar

Supplementary Information

Predicting treatment outcome using kinome activity profiling in HER2+ breast cancer biopsies

Donna O. Debets1,*, Erik L. de Graaf2,*, Marte C. Liefaard3, Gabe S. Sonke4,5, Esther H. Lips3 Anna Ressa2, and Maarten Altelaar1,#

1Biomolecular Mass Spectrometry and Proteomics, Bijvoet Center for Biomolecular Research and Utrecht Institute for Pharmaceutical Sciences, University of Utrecht, Utrecht, 3584 CH Utrecht, Netherlands.
2Pepscope B.V. Nieuwe Kanaal 7, 6709 PA Wageningen, the Netherlands.
3Department of Molecular Pathology, The Netherlands Cancer Institute, Amsterdam, the Netherlands.
4Department of Medical Oncology, The Netherlands Cancer Institute, Amsterdam, the Netherlands.
5Department of Medical Oncology, University of Amsterdam, Amsterdam, the Netherlands.

* Equal contributions
# Correspondence to: m.altelaar@uu.nl

Supplementary Table 1. Patient information

Patient	*Histology*	*Tumour grade*	*ER (%)*	*PR (%)*	*HER2 score*	*Treatment outcome*	*Tumour (%)*
1	ID	2	95	70	3+	pCR	80
2	ID	2	100	0	3+	pCR	80
3	ID	3	20	0	3+	pCR	80
4	ID	2	80	5	3+	pCR	70
5	IL	3	100	40	3+	npCR	80
6	ID	2	40	0	3+	pCR	60
7	ID	2	100	1	2+	pCR	70
9	ID	3	80	70	3+	npCR	70
10	ID	2	0	0	3+	pCR	70
*11A**	ID	2	0	0	3+	pCR	60
*11B**	ID	2	0	0	3+	pCR	60
12	ID	3	0	0	3+	pCR	70
13	ID	2	0	0	3+	pCR	60
14	ID	3	60	5	2+	npCR	60
15	ID	3	0	0	3+	pCR	60
16	IL	3	90	0	3+	pCR	80
17	ID	3	90	100	3+	npCR	70
18	ID	3	100	20	3+	No pCR	80
20	ID	3	90	40	3+	pCR	60
21	ID	3	99	1	2+	No pCR	80
22	ID	3	100	60	2+	No pCR	80
23	ID	2	70	70	3+	pCR	60
24	ID	3	10	0	2+	pCR	80
25	ID	3	100	100	3+	pCR	70
26	ID	2	50	100	3+	No pCR	70
27	ID	3	100	5	3+	No pCR	80
28	ID	3	100	90	3+	No pCR	70
30	ID	2	100	40	3+	No pCR	60
32	ID	3	100	1	3+	npCR	60
33	ID	3	100	0	2+	pCR	80
34	ID	2	100	0	3+	npCR	70
36	ID	3	100	5	3+	No pCR	80
37	IL	2	100	0	2+	No pCR	80

ID: invasive ductal
IL: invasive lobular
*Workflow replicate

Supplementary Figures

Supplementary Figure 1. Histological/H&E image representation of the three treatment outcome groups, related to Figure 1. Representative examples of pre-treatment HER2+ biopsies are shown, with respectively a pCR, npCR and no pCR post treatment. Pathology response measurement was done by a breast pathologist during the microscopic inspection of the surgical resection specimen at routine diagnostics.

Supplementary Figure 2, related to Figure 1. Characterisation of quantified T-loops and kinases in this study. A) Total number of quantified kinases, phosphopeptides, phosphosites and T-loops across all biopsies. B) Average kinase T-loop abundance of the endogenous signals, coloured by kinase family. Amongst the most highly abundant kinases are family members of the CMGC-family (GSK3A and CDKs). Kinase class abbreviations: CAMK, Ca 2+ /calmodulin-dependent kinase; STE, serine/threonine protein kinases; CMGC, CDK, MAPK, GSK and CDK-like protein kinases; AGC, protein kinase A, G, and C families; TKL, tyrosine kinase-like kinases. C) Boxplot of T-loop standard abundance per individual runs. D) Correlation plot of all quantified kinase T-loops in this study. Kinase names are coloured according to the kinase family. Pearson correlation was used. Hierarchical clustering method was Ward's. E) Kinase mutation frequency in breast cancer, reported by TCGA (The Cancer Genome Atlas). Many detected kinases show a very high mutation frequency in breast cancer, comparable to other well-known breast cancer associated genes such as BRCA and ESR1. F) Number of references in PhosphoSitePlus for each endogenously detected T-loop phosphosite. Although many (but not all) sites have been identified often in high throughput studies (HTS), the biological relevance has been mostly understudied, as evidenced by the low number of low througphut studies (LTS).

Supplementary Figure 3. Boxplots of kinase T-loops, related to Figure 3. A) Boxplots of differentially regulated kinase T-loops. * p-value < 0.05; ** p-value < 0.001. B) Boxplots of kinase T-loops of p38A that were not significantly changing between treatment outcome groups.

Supplementary Figure 4. Detected oxidised and non-oxidised kinase T-loops, related to Figure 3. A) Correlation plots, comparing the abundance of the oxidised and non-oxidised T-loop versions. Good linear correlations show the rate of oxidation is similar between samples. Moreover, oxidised versions are >10-fold lower in abundance. B) The number of quantified T loops is lower for the oxidised T loop phosphopeptides.

Supplementary Figure 1.

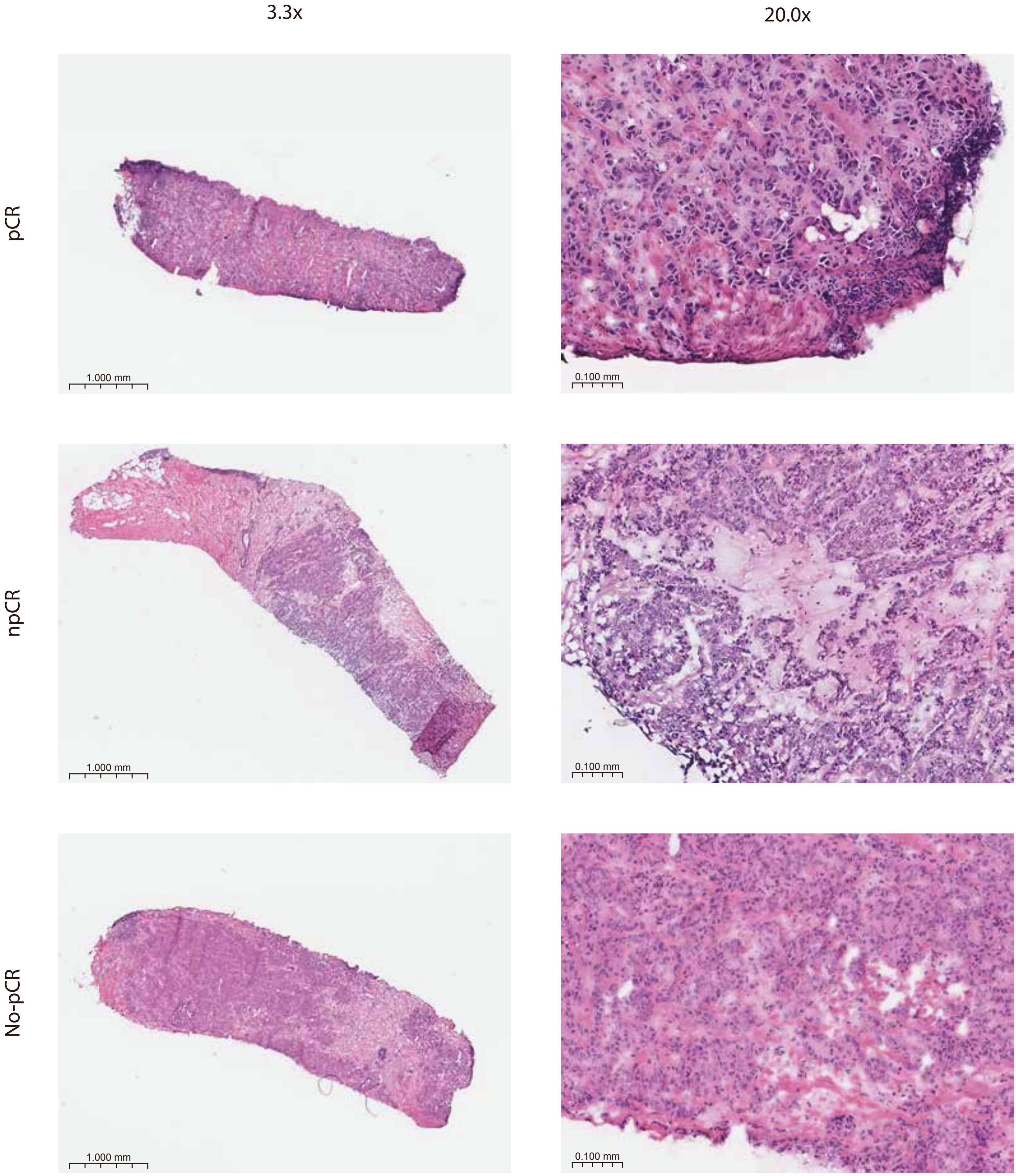

Supplementary Figure 2.

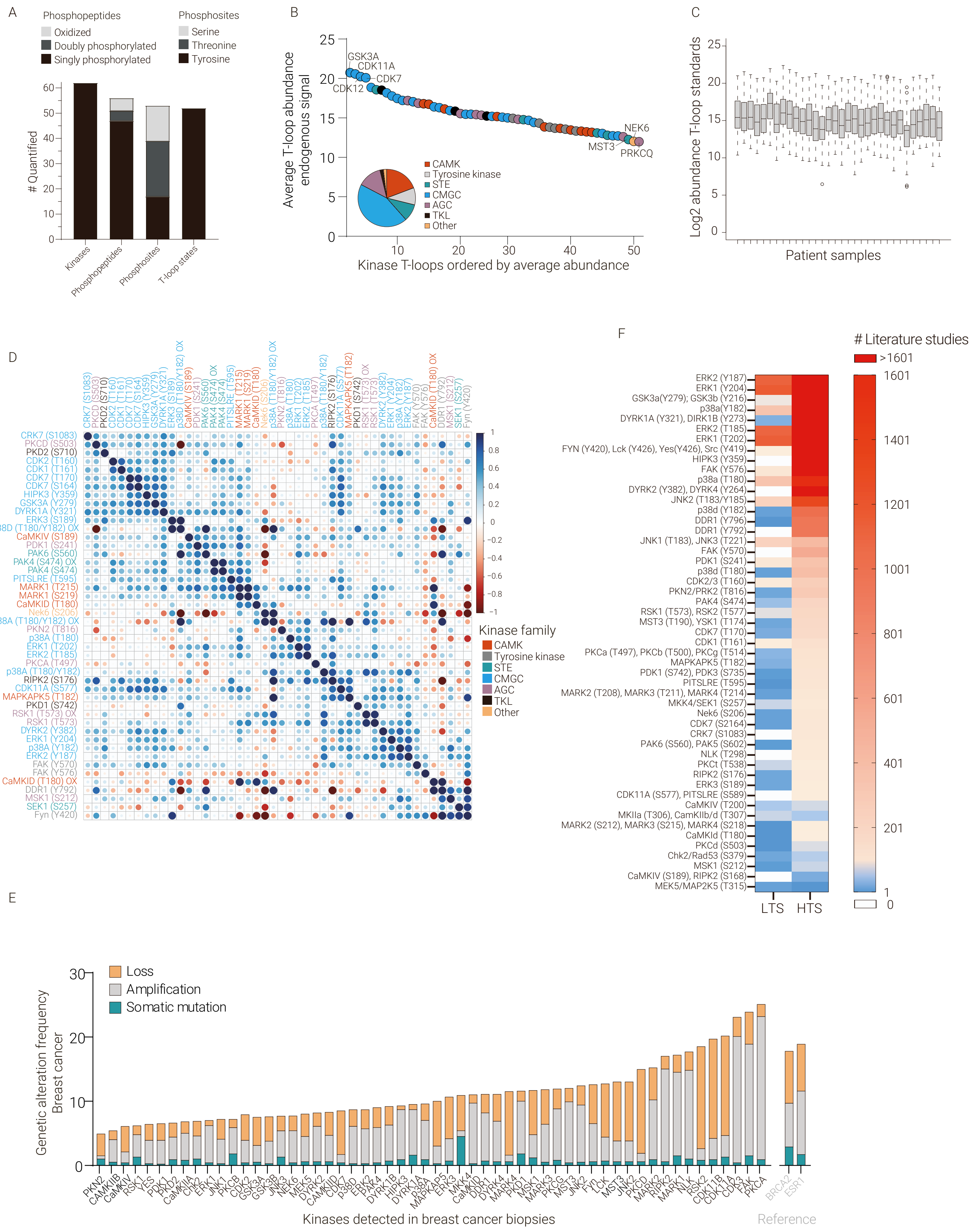

Supplementary Figure 3.

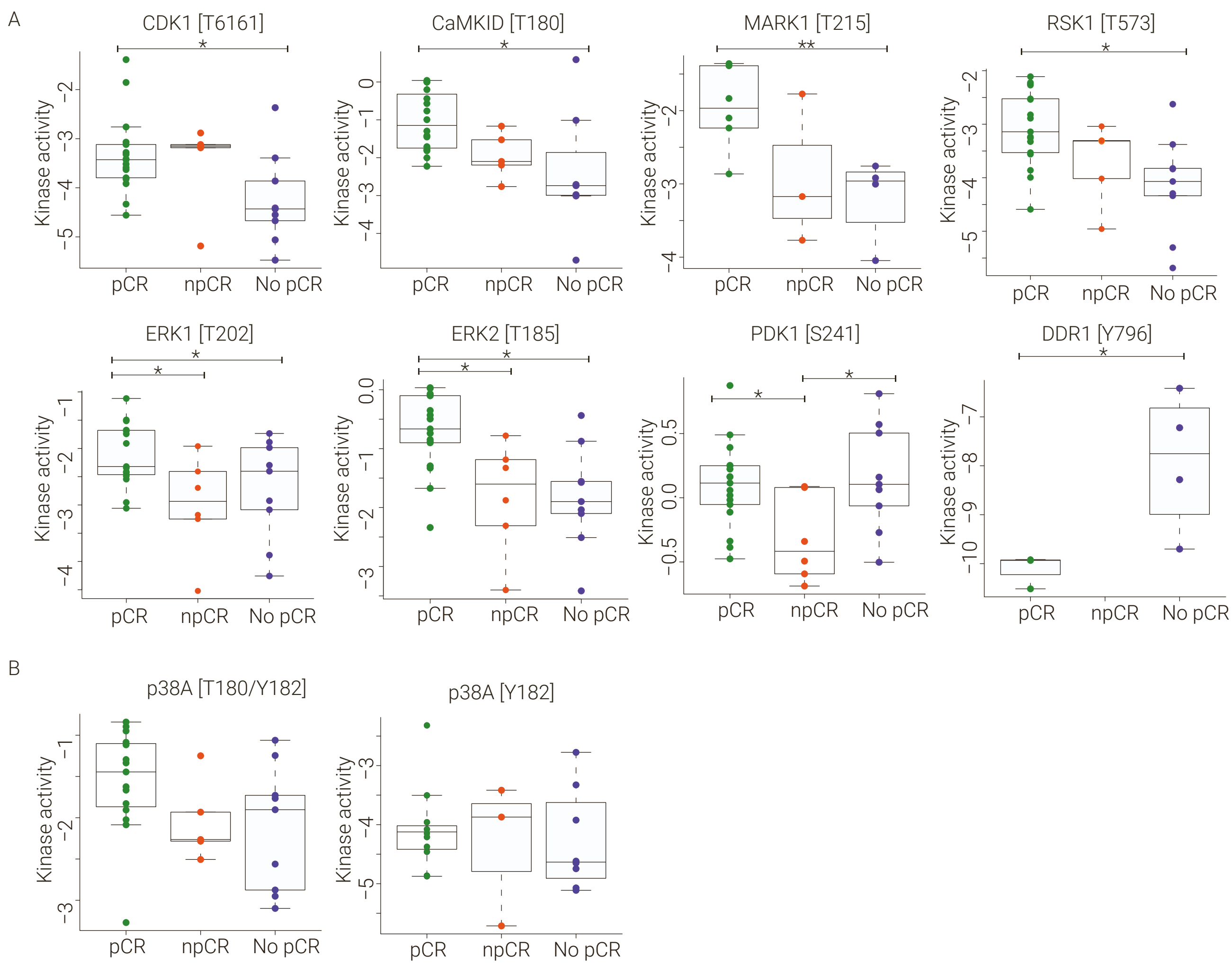

Supplementary Figure 4.

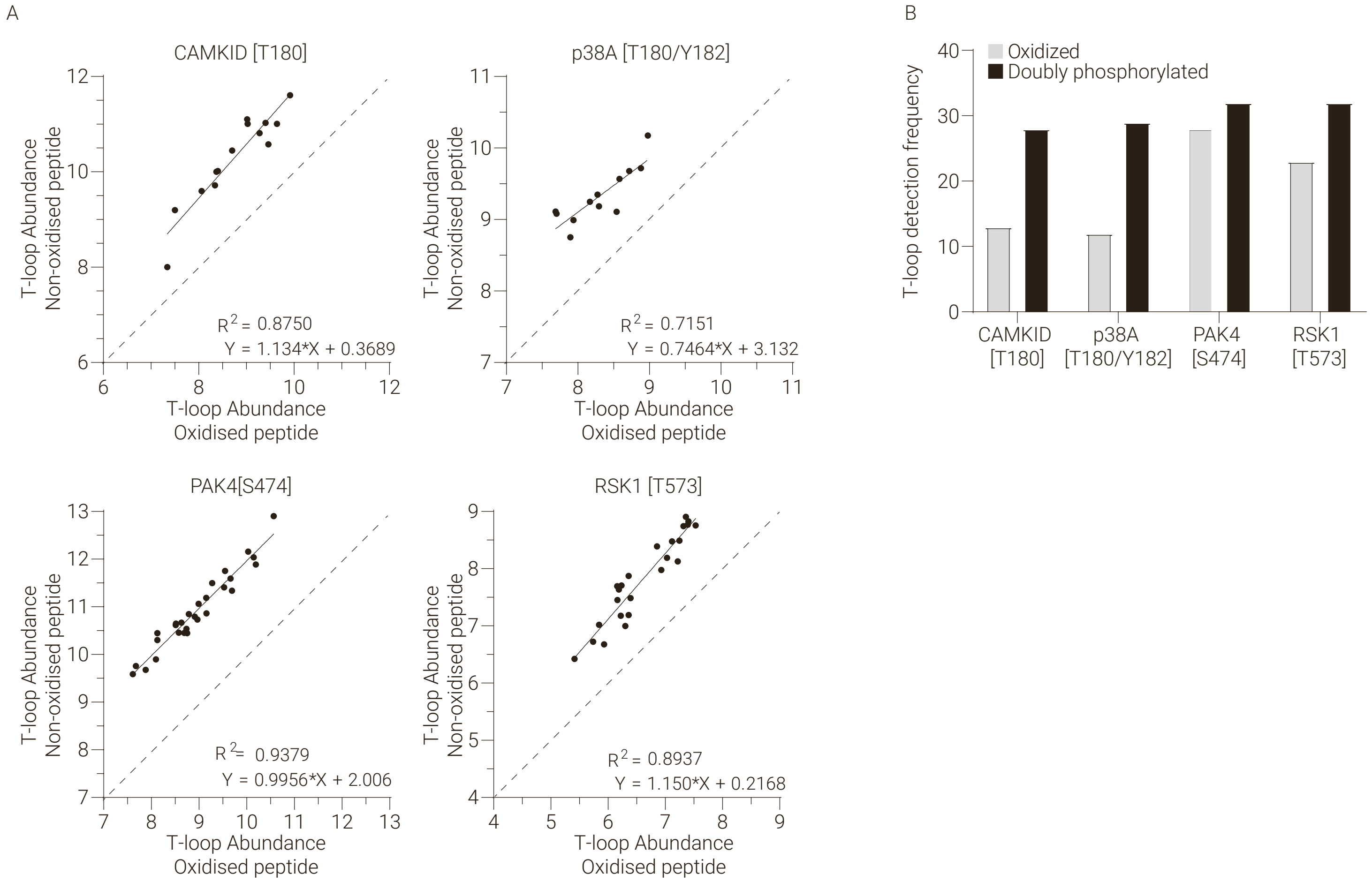

Supplement: Document S1. Figures S1‒S4 and Table S1 [file mmc1.pdf]
